# Supplementary material for: Investigating the Antituberculosis Activity of Selected Commercial Essential Oils and Identification of Active Constituents Using a Biochemometrics Approach and In Silico Modeling
Source: Antibiotics (Basel). 2022 Jul 14;11(7):948. doi: 10.3390/antibiotics11070948 (PMC9311982; doi:10.3390/antibiotics11070948)
Supplement: Supplementary file 1 [file antibiotics-11-00948-s001.zip › antibiotics-1808831-supplementary.pdf]

antibiotics

MDPI

Supplementary Material

Table S1. This is a table of the 85 essential oils studied, the major chemical compounds identified by GC-MS-FID, and the mean MIC values.

		MIC values (mg/mL)			MIC values (µg/mL)
**Scientific name**	**Major compounds (% area)**	***M. smegmatis***	***M. fortuitum***	***M. gordonae***	***M. tuberculosis***
Abies alba Mill.	**α-Pinene** (34.0), **limonene** (20.3), **β-pinene** (17.9), **camphene** (13.1), β-caryophyllene (5.8)	2.0	2.0	8.0	> 256
Abies balsamea (L.) Mill.	**β-Pinene** (34.7), 3-carene (15.2), **α-pinene** (12.1), bornyl acetate (9.3), **limonene** (6.9), β-phellandrene (6.8), **camphene** (6.2)	2.0	2.0	4.0	> 256
Amyris balsamifera L.	Kusenol (22.8), 7-epi-α-eudesmol (12.1), elemol (10.5), γ-eudesmol (9.8), epi-γ-eudesmol (8.3), β-eudesmol (7.2), α-eudesmol (7.1)	2.0	1.0	4.0	> 256
Apium graveolens var. *dulce* (Mill.) DC.	**Limonene** (77.2), selinene (14.6)	> 8.0	2.0	8.0	> 256
Artemisia herba-alba Asso	α-Thujone (64.5), camphor (15.7), β-thujone (10.6)	2.0	2.0	4.0	> 256
Canarium luzonicum (Blume) A. Gray	**Limonene** (51.1), elemol (12.7), α-phellandrene (10.8), elemicin (5.6)	> 8.0	**1.0**	4.0	> 256
Cedrelopsis grevei Baill. and Courchet	Ishwarane (23.3), α-copaene (9.4), β-elemene (9.2), α-ylangene (6.9), curcumene (5.7)	**1.0**	2.0	4.0	> 256
Cinnamomum camphora (L.) J. Presl (CT cineole)	**1,8-Cineole** (58.3), **sabinene** (12.7), α-terpineol (7.8), **α-pinene** (4.5)	**1.0**	**1.0**	> 8.0	> 256

Cinnamomum camphora (L.) J. Presl (CT linalool)	**Linalool** (99.1)	2.0	**1.0**	4.0	> 256
Cinnamomum cassia (L.) J. Presl	Cinnamaldehyde (81.2), 2-methoxycinnamaldehyde (12.0)	2.0	**0.50**	**1.0**	**4.00**
Cinnamomum zeylanicum Blume (bark)	Methyl methanthranilate (42.7), cinnamaldehyde (24.3), **γ-terpinene** (11.3), **limonene** (4.3)	**1.0**	**0.50**	**0.25**	**8.00**
Cinnamomum zeylanicum Blume (leaf)	Eugenol (83.0)	2.0	**1.0**	**1.0**	> 256
Citrus aurantium subsp. *amara* (Link) Engl.	**Linalool** (43.0), **limonene** (18.4), **β-pinene** (12.7), **γ-terpinene** (4.7), terpinyl acetate (4.5), nerolidol (4.0)	4.0	**1.0**	4.0	> 256
Citrus paradisi Macfad.	**Limonene** (83.7)	2.0	2.0	4.0	> 256
Citrus reticulata Blanco	**Limonene** (71.4), **γ-terpinene** (16.3)	> 8.0	2.0	4.0	> 256
Copaifera officinalis L.	β-Caryophyllene (58.8), α-humulene (7.1), γ-muurolene (7.1), α-copaene (5.3)	> 8.0	2.0	8.0	> 256
Coriandrum sativum L.	**Linalool** (40.0), decenol (18.4), decenal (14.1), decanal (5.6), **γ-terpinene** (4.3)	**1.0**	2.0	8.0	> 256
Crithmum maritimum L.	**γ-Terpinene** (45.4), β-phellandrene (16.3), thymol methyl ether (9.1), *p*-cymene (7.4), apiol (6.3), **sabinene** (4.2)	2.0	2.0	4.0	> 256
Cymbopogon citratus (DC.) Stapf	Geranial (47.5), neral (30.1), **limonene** (5.3)	2.0	**0.50**	**1.0**	> 256
Cymbopogon flexuosus (Nees ex Steud.) W. Watson	Geranial (49.5), neral (31.7)	**1.0**	**1.0**	2.0	256
Cymbopogon giganteus Chiov.	*Trans-p*-mentha-1(7),8-dien-2-ol (22.3), *trans-p*-mentha-2,8-dien-1-ol (19.8), *cis-p*-mentha-1(7),8-	2.0	**0.50**	**1.0**	> 256

	dien-2-ol (18.7), **limonene** (12.0), *cis-p*-mentha-2,8-dien-1-ol (9.9)				
Cymbopogon martinii var. *motia*	Citronellal (37.3), geraniol (23.4), citronellol (12.2)	2.0	**1.0**	4.0	> 256
Cymbopogon winterianus Jowitt ex Bor	Geraniol (81.9), geranyl butyrate (9.0)	**1.0**	**1.0**	**0.50**	> 256
Daucus carota L.	Carotol (47.6), ***α*-pinene** (8.6), β-caryophyllene (6.0)	**1.0**	2.0	8.0	> 256
Elettaria cardamomum (L.) Maton	Terpinyl acetate (43.9), **1,8-cineole** (32.0), linalyl acetate (5.5)	2.0	2.0	2.0	> 256
Eucalyptus citriodora Hook.	Citronellal (83.7)	4.0	**1.0**	4.0	> 256
Eucalyptus dives Schauer	Piperitone (42.9), *α*-phellandrene (20.6), **1,8-cineole** (10.9), terpinen-4-ol (5.4)	**1.0**	**1.0**	> 8.0	> 256
Eucalyptus globulus Labill.	**1,8-Cineole** (81.7), **limonene** (8.1)	> 8.0	2.0	> 8.0	> 256
Eucalyptus radiata A. Cunn. ex DC.	**1,8-Cineole** (69.5), *α*-terpineol (13.7), **limonene** (5.3)	2.0	2.0	> 8.0	> 256
Eucalyptus smithii F. Muell. ex R.T. Baker	**1,8-Cineole** (81.7), **limonene** (5.6)	4.0	**1.0**	> 8,0	> 256
Eugenia caryophyllus (Spreng.) Bullock and S.G. Harrison	Eugenol (81.5), isoeugenol (14.3)	2.0	**1.0**	**1.0**	> 256
Foeniculum vulgare Mill.	*Trans*-anethole (88.4)	2.0	4.0	8.0	> 256
Illicium verum Hook.f.	*Trans*-anethole (94.0)	2.0	4.0	2.0	> 256
Jasminum officinale L.	Benzyl acetate (32.1), isophytol (17.5), **linalool** (7.8), *cis*-jasmone (5.9), neophytadiene (5.4), indole (4.3)	**1.0**	**1.0**	2.0	256

Juniperus communis L.	***α*-Pinene** (38.4), **sabinene** (9.6), **γ-terpinene** (6.1), **limonene** (5.5), myrcene (4.2)	2.0	2.0	4.0	> 256
Juniperus communis var. *alpina* Suter	**Limonene** (28.5), β-phellandrene (16.7), terpinyl acetate (15.1), ***α*-pinene** (10.0), **sabinene** (4.5)	4.0	2.0	8.0	> 256
Juniperus oxycedrus L.	β-cadinene (32.1), cubenol+1-epi cubenol (15.6), β-caryophyllene (6.2), cadina-1(6),4-diene (5.9), *α*-caryophyllene (5.0)	4.0	**1.0**	4.0	> 256
Juniperus virginiana L.	Thujopsene (30.0), *α*-cedrene (25.2), cedrol (20.0), β-cedrene (5.4)	**1.0**	**1.0**	2.0	> 256
Laurus nobilis L.	**1,8-Cineole** (47.0), terpinyl acetate (15.0), **sabinene** (7.4), **β-pinene** (4.2), **linalool** (4.3), ***α*-pinene** (4.1)	**1.0**	2.0	4.0	> 256
Lavandula × burnatii Briq. clone *abrialis*	**Linalool** (38.9), linalyl acetate (21.5), camphor (4.2)	4.0	2.0	4.0	> 256
Lavandula × burnatii Briq. clone *super*	**Linalool** (40.8), linalyl acetate (38.9), camphor (4.7)	> 8.0	**1.0**	8.0	> 256
Lavandula angustifolia Mill.	**Linalool** (40.4), linalyl acetate (36.5), β-caryophyllene (4.3)	2.0	**1.0**	2.0	> 256
Lavandula stoechas L.	Camphor (32.8), frenchone (27.2), **1,8-cineole** (6.7), **camphene** (4.2)	2.0	2.0	4.0	> 256
Leptospermum petersonii F.M. Bailey	Geranial (37.2), neral (23.2), citronellal (20.8)	**1.0**	**1.0**	2.0	> 256
Levisticum officinale W.D.J. Koch	5-Heptylcyclohexa-1,3-diene (26.4), acetophenone (10.6), β-phellandrene (6.9), vinylguaiacol (6.5),	**0.50**	**0.50**	**0.50**	**4.00**

	terpinyl acetate (5.2), amyl benzene (5.0), furfural (4.6), terpinen-4-ol (4.0)				
Lippia citriodora (Palau) Kunth	**Limonene** (22.1), geranial (10.4), **1,8-cineole** (6.9), neral (6.9), β-caryophyllene (6.5)	2.0	2.0	2.0	> 256
Litsea citrata Blume	Ligustilide (54.4), geranial (18.0), neral (12.2), **limonene** (5.5)	2.0	**0.50**	2.0	> 256
Matricaria recutita L.	β-Farnesene (54.1), α-bisabolol oxide b (11.0), α-bisabolol (8.4), α-farnesene (8.2), γ-muurolene (5.6),	2.0	2.0	2.0	256
Melaleuca alternifolia (Maiden and Betche) Cheel	Terpinen-4-ol (45.7), **γ-terpinene** (20.6), α-terpinene (9.3)	2.0	**1.0**	2.0	> 256
Melaleuca cajuputi Powell	**1,8-Cineole** (72.2), **limonene** (6.7), α-terpineol (6.9)	4.0	4.0	4.0	> 256
Melaleuca quinquenervia (Cav.) S.T. Blake (CT cineole)	**1,8-Cineole** (59.2), α-terpineol (8.5), **limonene** (6.9), **α-pinene** (6.7), viridiflorol (5.4)	**1.0**	2.0	8.0	> 256
Mentha × *citrata* Ehrh.	Linalyl acetate (41.1), **linalool** (33.8)	2.0	2.0	4.0	> 256
Mentha × *piperita* L.	Menthol (32.6), menthone (32.2), menthyl acetate (5.5), **1,8-cineole** (5.5), isomenthone (4.8)	2.0	2.0	4.0	> 256
Mentha pulegium L.	Pulegone (87.7)	2.0	2.0	2.0	> 256
Mentha spicata L.	Carvone (61.6), carveol (13.8), **limonene** (12.6)	4.0	**1.0**	2.0	> 256
Myristica fragrans Houtt.	**α-Pinene** (22.1), **sabinene** (15.4), **β-pinene** (14.6), myristicin (10.4), terpinen-4-ol (8.2), **γ-terpinene** (5.0)	> 8.0	**1.0**	8.0	> 256
Myrtus communis L. (CT cineole)	**α-Pinene** (50.5), **1,8-cineole** (25.7), **limonene** (10.4)	> 8.0	**1.0**	**0.50**	> 256

Myrtus communis L. (CT myrtenyl acetaat)	**1,8-Cineole** (26.9), **α-pinene** (18.0), myrtenyl acetate (17.7), neryl acetate (11.2), **limonene** (9.8)	2.0	**1.0**	> 8.0	> 256
Nardostachys jatamansi (D. Don) DC.	β-Gurjunene (13.1), guaia-6,9-diene (7.0), jatamansone (6.0)	**1.0**	2.0	4.0	> 256
Ocimum basilicum L.	Estragole (74.0), **linalool** (19.6)	4.0	**1.0**	4.0	> 256
Origanum majorana L. (CT thujanol)	*Trans*-sabinene hydrate (23.8), terpinen-4-ol (23.5), **γ-terpinene** (10.3), α-terpinene (6.6), **sabinene** (6.4), *cis*-sabinene hydrate (4.2)	**1.0**	**1.0**	2.0	> 256
Origanum majorana L.	Terpinen-4-ol (28.5), *trans*-sabinene hydrate (17.0), **γ-terpinene** (13.3), α-terpinene (7.6), **sabinene** (6.8), *cis*-sabinene hydrate (4.6)	2.0	**1.0**	**1.0**	> 256
Pelargonium asperum Willd.	Citronellol (37.5), citronellyl formate (9.3), eudesmol (7.0), isomenthone (5.7),	2.0	**1.0**	2.0	> 256
Petroselinum crispum (Mill.) Fuss	α- Pinene (19.4), myristicine (15.2), **β-pinene** (14.6), β-phellandrene (8.8), p-mentha-1,3,8-triene (6.7), myrcene (4.8), allyltetramethoxybenzene (4.1)	2.0	2.0	2.0	> 256
Picea mariana (Mill.) Britton, Sterns and Poggenb.	Bornyl acetate (30.7), **camphene** (20.7), **α-pinene** (14.7), d-3-carene (6.8)	> 8.0	2.0	8.0	> 256
Pinus pinaster Aiton	**α-Pinene** (75.1), **β-pinene** (17.4)	> 8.0	**1.0**	8.0	> 256
Pinus ponderosa Douglas ex C. Lawson	**β-Pinene** (39.4), estragole (20.2), d-3-carene (18.6), **α-pinene** (8.7)	**1.0**	2.0	2.0	> 256
Pinus sylvestris L.	**α-Pinene** (40.6), **β-pinene** (25.7), **limonene** (8.9), myrcene (5.1)	2.0	2.0	8.0	> 256

Pogostemon cablin (Blanco) Benth.	Patchoulol (34.5), α-bulnesene (18.6), α-guaiene (14.8), seychellene (8.4), α-patchoulene (5.1)	**1.0**	2.0	8.0	256
Rosmarinus officinalis L. (CT cineole)	**1,8-Cineole** (48.7), camphor (11.9), **α-pinene** (10.7), **β-pinene** (6.1), β-caryophyllene (4.9)	4.0	**1.0**	4.0	> 256
Rosmarinus officinalis L. (CT kamfer)	**α-Pinene** (22.4), **1,8-cineole** (22.3), camphor (19.9), **camphene** (9.7), **limonene** (4.4)	4.0	**1.0**	2.0	> 256
Rosmarinus officinalis L. (CT verbenone)	**α-Pinene** (37.5), bornyl acetate (9.7), **camphene** (9.2), camphor (7.7), isoborneol (6.1), **1,8-cineole** (6.0), verbenone (4.4)	> 8.0	2.0	8.0	> 256
Salvia lavandulifolia Vahl	Camphor (33.5), **1,8-cineole** (29.0), **camphene** (6.5), **limonene** (4.7)	**1.0**	2.0	8.0	> 256
Salvia officinalis L.	α-Thujone (45.4), β-thujone (16.4), **1,8-cineole** (12.1), β-caryophyllene (5.4)	2.0	2.0	4.0	> 256
Salvia sclarea L.	Linalyl acetate (76.7), **linalool** (15.6)	> 8.0	2.0	8.0	> 256
Satureja hortensis L.	**γ-Terpinene** (39.3), carvacrol (34.7), *p*-cymene (11.9)	**1.0**	**1.0**	4.0	> 256
Satureja montana L.	Carvacrol (50.4), **γ-terpinene** (16.5), *p*-cymene (14.2)	**1.0**	**0.50**	2.0	> 256
Solidago canadensis L.	Bicyclosequiphellandrene (31.9), **α-pinene** (12.7), **limonene** (10.2), myrcene (7.4), **sabinene** (4.6), bornyl acetate (4.5)	> 8.0	2.0	8.0	> 256
Thuja occidentalis L.	α-Thujone (51.3), frenchone (16.4), β-thujone (7.2), benzyl benzoate (5.4), **sabinene** (4.0)	> 8.0	2.0	8.0	> 256

Thymus saturejoides Coss.	α-Terpineol (16.0), borneol (15.2), **camphene** (10.5), β-caryophyllene (10.2), thymol (9.3), **linalool** (6.3), **β-pinene** (5.7), *p*-cymene (5.5)	2.0	2.0	**1.0**	> 256
Thymus vulgaris L. (CT thymol)	Thymol (40.8), *p*-cymene (26.5), **γ-terpinene** (10.8)	2.0	**1.0**	**1.0**	> 256
Thymus zygis L.	Thymol (53.1), *p*-cymene (20.7), **γ-terpinene** (5.8), **linalool** (4.2)	**1.0**	**1.0**	2.0	> 256
Trachyspermum ammi (L.) Sprague	Thymol (37.8), **γ-terpinene** (33.8), *p*-cymene (22.3)	**1.0**	**1.0**	8.0	> 256
Tsuga canadensis (L.) Carrière	Bornyl acetate (34.8), **α-pinene** (19.2), **camphene** (15.0), tricyclene (5.9)	4.0	4.0	8.0	> 256
Valeriana officinalis L.	Bornyl acetate (39.9), **camphene** (23.0), **β-pinene** (4.1)	> 8.0	2.0	8.0	> 256
Rifampicin (mg/mL)		0.25	0.0125	0.50	0.004
Ciprofloxacin (mg/mL)		3.125	3.125	0.156	-
Ethambutol (μg/mL)		-	-	-	2.00
Streptomycin (μg/mL)		-	-	-	2.00
Isoniazid (μg/mL)		-	-	-	0.25

The most frequently occurring compounds and noteworthy MIC values are indicated in bold.

Table S2. The identified potential targets from using target identification algorithm implemented in CHEMBL. The entry corresponding to Mycobacterium tuberculosis is highlighted in bold.

Target	Predicted Target Name	Organism	Confidence 70%	Confidence 80%	Confidence 90%	Activity Threshold
CHEMBL1907591	Neuronal acetylcholine receptor; alpha4/beta4	Homo sapiens	empty	active	both	5
CHEMBL2590	Aminopeptidase N	Sus scrofa	empty	active	both	6
CHEMBL4244	Legumain	Homo sapiens	active	active	both	6.5
CHEMBL3891	Calpain 1	Homo sapiens	empty	active	both	6
CHEMBL2966	Adenosine deaminase	Bos taurus	active	active	active	6
CHEMBL4086	Prostanoid EP4 receptor	Rattus norvegicus	active	active	active	7
CHEMBL1899	Serotonin 3a (5-HT3a) receptor	Homo sapiens	active	active	active	5
CHEMBL2304402	Phosphodiesterase 5A	Canis lupus familiaris	empty	active	active	6.5
CHEMBL5568	Proto-oncogene tyrosine-protein kinase ROS	Homo sapiens	active	active	active	6.5
CHEMBL1907588	Acetylcholine receptor; alpha1/beta1/delta/gamma	Homo sapiens	active	active	active	5
CHEMBL2283	Carbonic anhydrase II	Bos taurus	inactive	both	both	6
CHEMBL4556	Glycine transporter 1	Rattus norvegicus	empty	both	both	6
CHEMBL4349	N-acylsphingosine-amidohydrolase	Homo sapiens	empty	both	both	6
CHEMBL2390810	Microtubule-associated protein 2	Homo sapiens	empty	empty	inactive	6
CHEMBL5062	Coagulation factor X	Oryctolagus cuniculus	empty	empty	empty	6.5
CHEMBL4793	Dipeptidyl peptidase IX	Homo sapiens	empty	empty	active	6
CHEMBL1907596	Neuronal acetylcholine receptor; alpha4/beta2	Rattus norvegicus	empty	empty	both	5
CHEMBL5077	Butyrylcholinesterase	Equus caballus	empty	empty	both	6

CHEMBL1944	Neprilysin	Homo sapiens	empty	empty	inactive	6
CHEMBL1907594	Neuronal acetylcholine receptor; alpha3/beta4	Homo sapiens	empty	empty	both	5
CHEMBL1907595	Neuronal acetylcholine receptor; alpha4/beta4	Rattus norvegicus	empty	empty	inactive	5
CHEMBL3199	Acetylcholinesterase	Rattus norvegicus	empty	empty	both	6
CHEMBL3589	Adenosine kinase	Homo sapiens	empty	empty	both	6
CHEMBL3403	Butyrylcholinesterase	Rattus norvegicus	empty	empty	inactive	6
CHEMBL4653	Dipeptidyl peptidase IV	Rattus norvegicus	empty	empty	inactive	6
CHEMBL278	Integrin alpha-4	Homo sapiens	empty	empty	empty	6.5
CHEMBL4336	Prostanoid EP3 receptor	Mus musculus	empty	empty	inactive	7
CHEMBL3922	Methionine aminopeptidase 2	Homo sapiens	empty	empty	both	6
CHEMBL5163	Sodium channel protein type III alpha subunit	Homo sapiens	empty	empty	inactive	5
CHEMBL2096675	Integrin alpha-V/beta-5	Homo sapiens	empty	empty	both	6
CHEMBL4657	Dipeptidyl peptidase VIII	Homo sapiens	empty	empty	active	6
CHEMBL2411	Serotonin 3a (5-HT3a) receptor	Rattus norvegicus	empty	empty	inactive	6.5
CHEMBL2363	Dihydrofolate reductase	Rattus norvegicus	empty	empty	both	6
CHEMBL2304404	Adenosine A1 receptor	Cavia porcellus	empty	empty	both	6
CHEMBL311	Glutamate [NMDA] receptor subunit epsilon 2	Rattus norvegicus	empty	empty	empty	5
CHEMBL276	Muscarinic acetylcholine receptor M1	Rattus norvegicus	empty	empty	active	7
CHEMBL2575	Dihydrofolate reductase	Gallus gallus	empty	empty	inactive	6
CHEMBL3085613	DNA gyrase subunit B	Mycobacterium smegmatis	empty	empty	inactive	6
CHEMBL1782	Farnesyl diphosphate synthase	Homo sapiens	empty	empty	inactive	6

CHEMBL2288	Peptidyl-prolyl cis-trans isomerase NIMA-interacting 1	Homo sapiens	empty	empty	both	6
CHEMBL5457	Dihydrofolate reductase	Mycobacterium avium	empty	empty	empty	6
CHEMBL1947	Thyroid hormone receptor beta-1	Homo sapiens	empty	inactive	inactive	7
CHEMBL226	Adenosine A1 receptor	Homo sapiens	inactive	inactive	inactive	7
CHEMBL4618	Leukotriene A4 hydrolase	Homo sapiens	inactive	inactive	inactive	6
CHEMBL1827	Phosphodiesterase 5A	Homo sapiens	inactive	inactive	inactive	6
CHEMBL3771	Human herpes virus 5 capsid protein P40	Human cytomegalovirus (strain AD169) (HHV-5) (Human herpesvirus 5)	inactive	inactive	inactive	6
CHEMBL5658	Prostaglandin E synthase	Homo sapiens	inactive	inactive	inactive	6
CHEMBL3229	Anandamide amidohydrolase	Rattus norvegicus	inactive	inactive	inactive	6
CHEMBL4234	Estradiol 17-beta-dehydrogenase 3	Homo sapiens	inactive	inactive	inactive	6
CHEMBL4552	Peripheral-type benzodiazepine receptor	Rattus norvegicus	inactive	inactive	inactive	6
CHEMBL5103	Histone deacetylase 10	Homo sapiens	inactive	inactive	inactive	6
CHEMBL4779	Papain	Carica papaya	inactive	inactive	inactive	6
CHEMBL2334	Caspase-3	Homo sapiens	inactive	inactive	inactive	6
CHEMBL5498	Muscarinic acetylcholine receptor M3	Cavia porcellus	inactive	inactive	inactive	7
CHEMBL233	Mu opioid receptor	Homo sapiens	inactive	inactive	inactive	7
CHEMBL1991	Inhibitor of nuclear factor kappa B kinase beta subunit	Homo sapiens	inactive	inactive	inactive	7.5

CHEMBL4816	Serine/threonine-protein kinase AKT3	Homo sapiens	inactive	inactive	inactive	7.5
CHEMBL2056	Dopamine D1 receptor	Homo sapiens	inactive	inactive	inactive	7
CHEMBL5936	Toll-like receptor 7	Homo sapiens	inactive	inactive	inactive	6
CHEMBL231	Histamine H1 receptor	Homo sapiens	inactive	inactive	inactive	7
CHEMBL219	Dopamine D4 receptor	Homo sapiens	inactive	inactive	inactive	7
CHEMBL2487	Beta amyloid A4 protein	Homo sapiens	empty	inactive	inactive	6
CHEMBL1824	Receptor protein-tyrosine kinase erbB-2	Homo sapiens	inactive	inactive	inactive	7.5
CHEMBL3969	Carbonic anhydrase VB	Homo sapiens	inactive	inactive	inactive	6
CHEMBL2652	Phosphodiesterase 2A	Homo sapiens	inactive	inactive	inactive	6
CHEMBL3202	Prolyl endopeptidase	Homo sapiens	inactive	inactive	inactive	6
CHEMBL3571	Cannabinoid CB1 receptor	Rattus norvegicus	inactive	inactive	inactive	7
CHEMBL4550	5-lipoxygenase activating protein	Homo sapiens	inactive	inactive	inactive	6
CHEMBL3798	Calcitonin gene-related peptide type 1 receptor	Homo sapiens	inactive	inactive	inactive	7
CHEMBL218	Cannabinoid CB1 receptor	Homo sapiens	inactive	inactive	inactive	7
CHEMBL2243	Anandamide amidohydrolase	Homo sapiens	inactive	inactive	inactive	6
CHEMBL224	Serotonin 2a (5-HT2a) receptor	Homo sapiens	inactive	inactive	inactive	7
CHEMBL6031	Histone-lysine N-methyltransferase, H3 lysine-9 specific 5	Homo sapiens	inactive	inactive	inactive	6
CHEMBL4801	Caspase-1	Homo sapiens	inactive	inactive	inactive	6
CHEMBL1951	Monoamine oxidase A	Homo sapiens	inactive	inactive	inactive	6
CHEMBL4430	Cytochrome P450 17A1	Rattus norvegicus	inactive	inactive	inactive	6
CHEMBL4342	Group X secretory phospholipase A2	Homo sapiens	inactive	inactive	inactive	6

CHEMBL2111432	PI3-kinase p110-delta/p85-alpha	Homo sapiens	inactive	inactive	inactive	6
CHEMBL4408	Phosphodiesterase 8B	Homo sapiens	inactive	inactive	inactive	6
CHEMBL1941	Histamine H2 receptor	Homo sapiens	inactive	inactive	inactive	6.5
CHEMBL208	Progesterone receptor	Homo sapiens	inactive	inactive	inactive	7
CHEMBL220	Acetylcholinesterase	Homo sapiens	inactive	inactive	inactive	6
CHEMBL234	Dopamine D3 receptor	Homo sapiens	inactive	inactive	inactive	7
CHEMBL1835	Thromboxane-A synthase	Homo sapiens	inactive	inactive	inactive	6
CHEMBL3788	Serine/threonine-protein kinase PLK4	Homo sapiens	inactive	inactive	inactive	7.5
CHEMBL4018	Neuropeptide Y receptor type 2	Homo sapiens	inactive	inactive	inactive	7
CHEMBL2327	Neurokinin 2 receptor	Homo sapiens	inactive	inactive	inactive	7
CHEMBL5689	Tryptophan 5-hydroxylase 1	Homo sapiens	inactive	inactive	inactive	6
CHEMBL2695	Focal adhesion kinase 1	Homo sapiens	inactive	inactive	inactive	7.5
CHEMBL2326	Carbonic anhydrase VII	Homo sapiens	inactive	inactive	inactive	6
CHEMBL3776	Caspase-8	Homo sapiens	inactive	inactive	inactive	6
CHEMBL3004	Multidrug resistance-associated protein 1	Homo sapiens	inactive	inactive	inactive	6
CHEMBL4780	Acetylcholinesterase	Torpedo californica	inactive	inactive	inactive	6
CHEMBL4794	Vanilloid receptor	Homo sapiens	inactive	inactive	inactive	5
CHEMBL1808	Angiotensin-converting enzyme	Homo sapiens	inactive	inactive	inactive	6
CHEMBL3979	Peroxisome proliferator-activated receptor delta	Homo sapiens	inactive	inactive	inactive	7
CHEMBL235	Peroxisome proliferator-activated receptor gamma	Homo sapiens	inactive	inactive	inactive	7
CHEMBL1983	Serotonin 1d (5-HT1d) receptor	Homo sapiens	inactive	inactive	inactive	7
CHEMBL1798	Cysteinyl leukotriene receptor 1	Homo sapiens	inactive	inactive	inactive	7
CHEMBL2736	Metabotropic glutamate receptor 4	Homo sapiens	inactive	inactive	inactive	7

CHEMBL1942	Alpha-2b adrenergic receptor	Homo sapiens	inactive	inactive	inactive	7
CHEMBL237	Kappa opioid receptor	Homo sapiens	inactive	inactive	inactive	7
CHEMBL223	Alpha-1d adrenergic receptor	Homo sapiens	inactive	inactive	inactive	7
CHEMBL1822	Inosine-5'-monophosphate dehydrogenase 1	Homo sapiens	inactive	inactive	both	6
CHEMBL1836	Prostanoid EP4 receptor	Homo sapiens	inactive	inactive	inactive	7
CHEMBL3012	Phosphodiesterase 7A	Homo sapiens	inactive	inactive	inactive	6
CHEMBL5113	Orexin receptor 1	Homo sapiens	inactive	inactive	inactive	7
CHEMBL3563	Cruzipain	Trypanosoma cruzi	inactive	inactive	inactive	6
CHEMBL3991	Coagulation factor VII	Homo sapiens	inactive	inactive	inactive	6
CHEMBL1163125	Bromodomain-containing protein 4	Homo sapiens	inactive	inactive	inactive	6
CHEMBL4768	Acetylcholinesterase	Bos taurus	inactive	inactive	inactive	6
CHEMBL3952	Kappa opioid receptor	Cavia porcellus	inactive	inactive	inactive	7
CHEMBL3629	Casein kinase II alpha	Homo sapiens	inactive	inactive	inactive	7.5
CHEMBL222	Norepinephrine transporter	Homo sapiens	inactive	inactive	inactive	6
CHEMBL236	Delta opioid receptor	Homo sapiens	inactive	inactive	inactive	7
CHEMBL4608	Melanocortin receptor 5	Homo sapiens	inactive	inactive	inactive	7
CHEMBL4191	Monoglyceride lipase	Homo sapiens	inactive	inactive	inactive	6
CHEMBL4422	Free fatty acid receptor 1	Homo sapiens	inactive	inactive	inactive	7
CHEMBL3371	Serotonin 6 (5-HT6) receptor	Homo sapiens	inactive	inactive	inactive	7
CHEMBL5983	Aldo-keto reductase family 1 member B10	Homo sapiens	inactive	inactive	inactive	6
CHEMBL2034	Glucocorticoid receptor	Homo sapiens	inactive	inactive	inactive	7
CHEMBL286	Renin	Homo sapiens	inactive	inactive	inactive	6
CHEMBL4860	Apoptosis regulator Bcl-2	Homo sapiens	inactive	inactive	inactive	5
CHEMBL251	Adenosine A2a receptor	Homo sapiens	inactive	inactive	inactive	7

CHEMBL245	Muscarinic acetylcholine receptor M3	Homo sapiens	inactive	inactive	inactive	7
CHEMBL1850	Dopamine D5 receptor	Homo sapiens	inactive	inactive	inactive	7
CHEMBL2007625	Isocitrate dehydrogenase [NADP] cytoplasmic	Homo sapiens	inactive	inactive	inactive	6
CHEMBL2366505	Integrase	Human immunodeficiency virus 1	inactive	inactive	inactive	6
CHEMBL325	Histone deacetylase 1	Homo sapiens	inactive	inactive	inactive	6
CHEMBL3048	Nitric-oxide synthase, brain	Rattus norvegicus	inactive	inactive	inactive	6
CHEMBL319	Alpha-1a adrenergic receptor	Rattus norvegicus	inactive	inactive	inactive	7
CHEMBL3706	ADAM17	Homo sapiens	inactive	inactive	inactive	6
CHEMBL2163176	Lysine-specific demethylase 5C	Homo sapiens	empty	inactive	inactive	6
CHEMBL4530	Coagulation factor XIII	Homo sapiens	inactive	inactive	inactive	6
CHEMBL2095178	Protein farnesyltransferase	Bos taurus	inactive	inactive	inactive	6.5
CHEMBL1795101	Peptide deformylase	Escherichia coli	inactive	inactive	inactive	6
CHEMBL4040	MAP kinase ERK2	Homo sapiens	inactive	inactive	inactive	7.5
CHEMBL244	Coagulation factor X	Homo sapiens	inactive	inactive	inactive	6
CHEMBL4685	Indoleamine 2,3-dioxygenase	Homo sapiens	inactive	inactive	inactive	6
CHEMBL287	Sigma opioid receptor	Homo sapiens	inactive	inactive	inactive	6
CHEMBL3471	Human immunodeficiency virus type 1 integrase	Human immunodeficiency virus 1	inactive	inactive	inactive	6
CHEMBL2035	Muscarinic acetylcholine receptor M5	Homo sapiens	inactive	inactive	inactive	7
CHEMBL3459	Serotonin 1b (5-HT1b) receptor	Rattus norvegicus	inactive	inactive	inactive	6.5
CHEMBL5028	ADAM10	Homo sapiens	empty	inactive	both	6
CHEMBL3473	C-C chemokine receptor type 3	Homo sapiens	inactive	inactive	inactive	7

CHEMBL285	Acyl coenzyme A:cholesterol acyltransferase 1	Rattus norvegicus	inactive	inactive	inactive	6
CHEMBL4687	Plasmepsin 1	Plasmodium falciparum	inactive	inactive	inactive	6
CHEMBL4644	Melanocortin receptor 3	Homo sapiens	inactive	inactive	inactive	7
CHEMBL246	Beta-3 adrenergic receptor	Homo sapiens	inactive	inactive	inactive	7
CHEMBL252	Endothelin receptor ET-A	Homo sapiens	inactive	inactive	inactive	7
CHEMBL332	Matrix metalloproteinase-1	Homo sapiens	inactive	inactive	inactive	6
CHEMBL326	Alpha-1d adrenergic receptor	Rattus norvegicus	inactive	inactive	inactive	7
CHEMBL4718	MAP kinase-interacting serine/threonine-protein kinase MNK1	Homo sapiens	inactive	inactive	both	6.5
CHEMBL5406	Histone-arginine methyltransferase CARM1	Homo sapiens	empty	inactive	inactive	6
CHEMBL4282	Serine/threonine-protein kinase AKT	Homo sapiens	inactive	inactive	inactive	7.5
CHEMBL3710	Prostanoid EP3 receptor	Homo sapiens	inactive	inactive	inactive	7
CHEMBL5375	Hepatitis C virus NS5B RNA-dependent RNA polymerase	Hepatitis C virus	inactive	inactive	inactive	6
CHEMBL333	Matrix metalloproteinase-2	Homo sapiens	inactive	inactive	inactive	6
CHEMBL2208	MAP kinase-activated protein kinase 2	Homo sapiens	inactive	inactive	inactive	7.5
CHEMBL3880	Heat shock protein HSP 90-alpha	Homo sapiens	inactive	inactive	inactive	6
CHEMBL4123	Neurotensin receptor 1	Homo sapiens	inactive	inactive	inactive	7
CHEMBL1926	Dihydrofolate reductase	Pneumocystis carinii	inactive	inactive	inactive	6
CHEMBL284	Dipeptidyl peptidase IV	Homo sapiens	inactive	inactive	inactive	6
CHEMBL5017	Serotonin 4 (5-HT4) receptor	Cavia porcellus	inactive	inactive	inactive	7

CHEMBL243	Human immunodeficiency virus type 1 protease	Human immunodeficiency virus 1	inactive	inactive	inactive	6
CHEMBL3884	Sodium/glucose cotransporter 2	Homo sapiens	inactive	inactive	inactive	6
CHEMBL2366517	Protease	Human immunodeficiency virus 1	inactive	inactive	inactive	6
CHEMBL1856	Steroid 5-alpha-reductase 2	Homo sapiens	inactive	inactive	inactive	6.5
CHEMBL3933	Prostanoid DP receptor	Mus musculus	inactive	inactive	inactive	7
CHEMBL3072	Androgen Receptor	Rattus norvegicus	inactive	inactive	inactive	7
CHEMBL6136	Lysine-specific histone demethylase 1	Homo sapiens	inactive	inactive	inactive	6
CHEMBL1881	Prostanoid EP2 receptor	Homo sapiens	inactive	inactive	inactive	7
CHEMBL2095194	Coagulation factor VII/tissue factor	Homo sapiens	inactive	inactive	inactive	6
CHEMBL4523	Serine/threonine-protein kinase PIM2	Homo sapiens	inactive	inactive	inactive	7.5
CHEMBL2147	Serine/threonine-protein kinase PIM1	Homo sapiens	inactive	inactive	inactive	7.5
CHEMBL2366516	Reverse transcriptase	Human immunodeficiency virus 1	inactive	inactive	inactive	6
CHEMBL256	Adenosine A3 receptor	Homo sapiens	inactive	inactive	inactive	7
CHEMBL4132	Voltage-gated L-type calcium channel alpha-1D subunit	Rattus norvegicus	inactive	inactive	inactive	5
CHEMBL3649	Xanthine dehydrogenase	Bos taurus	inactive	inactive	inactive	6
CHEMBL242	Estrogen receptor beta	Homo sapiens	inactive	inactive	inactive	7
CHEMBL2094116	Serotonin 3 (5-HT3) receptor	Rattus norvegicus	empty	inactive	inactive	6.5

CHEMBL4330	Cysteinyl leukotriene receptor 2	Homo sapiens	inactive	inactive	inactive	7
CHEMBL5979	Alkaline phosphatase, tissue-nonspecific isozyme	Homo sapiens	inactive	inactive	both	6
CHEMBL3105	Poly [ADP-ribose] polymerase-1	Homo sapiens	inactive	inactive	inactive	6
CHEMBL268	Cathepsin K	Homo sapiens	inactive	inactive	inactive	6
CHEMBL254	Phosphodiesterase 4A	Homo sapiens	inactive	inactive	inactive	6
CHEMBL3717	Hepatocyte growth factor receptor	Homo sapiens	inactive	inactive	inactive	7.5
CHEMBL4078	Acetylcholinesterase	Electrophorus electricus	empty	inactive	inactive	6
CHEMBL1795139	Transmembrane protease serine 6	Homo sapiens	inactive	inactive	inactive	6
CHEMBL2186	Carbonic anhydrase XIII	Mus musculus	inactive	inactive	both	6
CHEMBL3501	Cholecystokinin A receptor	Cavia porcellus	inactive	inactive	inactive	7
CHEMBL2145	Kynurenine 3-monooxygenase	Homo sapiens	inactive	inactive	inactive	6
CHEMBL2622	Aldose reductase	Rattus norvegicus	inactive	inactive	inactive	6
CHEMBL4247	ALK tyrosine kinase receptor	Homo sapiens	inactive	inactive	inactive	7.5
CHEMBL335	Protein-tyrosine phosphatase 1B	Homo sapiens	inactive	inactive	inactive	6
CHEMBL6120	Solute carrier family 22 member 12	Homo sapiens	inactive	inactive	inactive	6
CHEMBL4051	Cystic fibrosis transmembrane conductance regulator	Homo sapiens	inactive	inactive	both	5
CHEMBL2434	Interleukin-8 receptor B	Homo sapiens	inactive	inactive	inactive	7
CHEMBL321	Matrix metalloproteinase 9	Homo sapiens	inactive	inactive	inactive	6
CHEMBL309	Muscarinic acetylcholine receptor M2	Rattus norvegicus	inactive	inactive	both	7
CHEMBL5373	Cannabinoid CB2 receptor	Mus musculus	inactive	inactive	inactive	7

CHEMBL1868	Vascular endothelial growth factor receptor 1	Homo sapiens	inactive	inactive	inactive	7.5
CHEMBL3892	Sphingosine 1-phosphate receptor Edg-3	Homo sapiens	inactive	inactive	inactive	7
CHEMBL255	Adenosine A2b receptor	Homo sapiens	inactive	inactive	inactive	7
CHEMBL269	Delta opioid receptor	Rattus norvegicus	inactive	inactive	inactive	7
CHEMBL3474	Phospholipase A2 group IIA	Homo sapiens	inactive	inactive	inactive	6
CHEMBL5763	Cholinesterase	Equus caballus	empty	inactive	inactive	6
CHEMBL3883328	Casein kinase II alpha'/ beta	Homo sapiens	inactive	inactive	inactive	6.5
CHEMBL2959	Tyrosine-protein kinase ITK/TSK	Homo sapiens	inactive	inactive	inactive	7.5
CHEMBL3486	Dihydroorotate dehydrogenase	Plasmodium falciparum	inactive	inactive	inactive	6
CHEMBL2954	Cathepsin S	Homo sapiens	inactive	inactive	inactive	6
CHEMBL4699	Isoprenylcysteine carboxyl methyltransferase	Homo sapiens	inactive	inactive	inactive	6
CHEMBL258	Tyrosine-protein kinase LCK	Homo sapiens	inactive	inactive	inactive	7.5
CHEMBL1865	Histone deacetylase 6	Homo sapiens	inactive	inactive	inactive	6
CHEMBL1871	Androgen Receptor	Homo sapiens	inactive	inactive	inactive	7
CHEMBL4074	Angiotensin-converting enzyme	Oryctolagus cuniculus	inactive	inactive	inactive	6
CHEMBL338	Dopamine transporter	Rattus norvegicus	empty	inactive	inactive	6
CHEMBL5815	C-C chemokine receptor type 9	Homo sapiens	inactive	inactive	inactive	7
CHEMBL2820	Coagulation factor XI	Homo sapiens	empty	inactive	inactive	6
CHEMBL3242	Carbonic anhydrase XII	Homo sapiens	inactive	inactive	inactive	6
CHEMBL5141	Cytochrome P450 26A1	Homo sapiens	inactive	inactive	inactive	6
CHEMBL259	Melanocortin receptor 4	Homo sapiens	inactive	inactive	inactive	7
CHEMBL2564	Metabotropic glutamate receptor 5	Rattus norvegicus	inactive	inactive	inactive	7

CHEMBL5747	CREB-binding protein	Homo sapiens	inactive	inactive	inactive	6
CHEMBL4465	Acyl coenzyme A:cholesterol acyltransferase 2	Homo sapiens	inactive	inactive	inactive	6
CHEMBL2000	Plasma kallikrein	Homo sapiens	inactive	inactive	inactive	6
CHEMBL2955	Sphingosine 1-phosphate receptor Edg-5	Homo sapiens	inactive	inactive	inactive	7
CHEMBL3038482	DNA gyrase	Staphylococcus aureus	inactive	inactive	inactive	6
CHEMBL2016	Coagulation factor IX	Homo sapiens	inactive	inactive	inactive	6
CHEMBL2002	Inosine-5'-monophosphate dehydrogenase 2	Homo sapiens	empty	inactive	inactive	6
CHEMBL5023	p53-binding protein Mdm-2	Homo sapiens	inactive	inactive	inactive	6
CHEMBL298	Cholecystokinin B receptor	Homo sapiens	inactive	inactive	inactive	7
CHEMBL273	Serotonin 1a (5-HT1a) receptor	Rattus norvegicus	inactive	inactive	inactive	7
CHEMBL3650	Fibroblast growth factor receptor 1	Homo sapiens	inactive	inactive	inactive	7.5
CHEMBL313	Serotonin transporter	Rattus norvegicus	inactive	inactive	inactive	6
CHEMBL3132741	Peregrin	Homo sapiens	inactive	inactive	inactive	6
CHEMBL2095172	GABA-A receptor; alpha-1/beta-2/gamma-2	Homo sapiens	inactive	inactive	inactive	5
CHEMBL2010636	Class 1 phosphodiesterase PDEB1	Trypanosoma brucei	inactive	inactive	inactive	6
CHEMBL1898	Serotonin 1b (5-HT1b) receptor	Homo sapiens	inactive	inactive	inactive	7
CHEMBL4062	Calpain 1	Sus scrofa	inactive	inactive	inactive	6
CHEMBL1867	Alpha-2a adrenergic receptor	Homo sapiens	inactive	inactive	inactive	7
CHEMBL299	Protein kinase C alpha	Homo sapiens	inactive	inactive	inactive	7.5
CHEMBL1921666	DNA gyrase subunit B	Staphylococcus aureus	inactive	inactive	inactive	6
CHEMBL5973	Carbonic anhydrase 15	Mus musculus	inactive	inactive	inactive	6
CHEMBL1075317	WD repeat-containing protein 5	Homo sapiens	inactive	inactive	inactive	6

CHEMBL4462	NAD-dependent deacetylase sirtuin 2	Homo sapiens	inactive	inactive	inactive	6
CHEMBL262	Glycogen synthase kinase-3 beta	Homo sapiens	inactive	inactive	inactive	7.5
CHEMBL4072	Cathepsin B	Homo sapiens	inactive	inactive	inactive	6
CHEMBL302	Adenosine A2a receptor	Rattus norvegicus	inactive	inactive	inactive	7
CHEMBL3286	Urokinase-type plasminogen activator	Homo sapiens	inactive	inactive	inactive	6
CHEMBL5185	Glutamate racemase	Helicobacter pylori J99	inactive	inactive	inactive	6.5
**CHEMBL1908385**	**Serine/threonine-protein kinase pknB**	**Mycobacterium tuberculosis**	**empty**	**inactive**	**both**	**6.5**
CHEMBL1889	Vasopressin V1a receptor	Homo sapiens	inactive	inactive	inactive	7
CHEMBL1916	Alpha-2c adrenergic receptor	Homo sapiens	inactive	inactive	inactive	7
CHEMBL288	Phosphodiesterase 4D	Homo sapiens	inactive	inactive	inactive	6
CHEMBL2789	Estradiol 17-beta-dehydrogenase 2	Homo sapiens	inactive	inactive	inactive	6
CHEMBL1914	Butyrylcholinesterase	Homo sapiens	inactive	inactive	inactive	6
CHEMBL2094135	Gamma-secretase	Homo sapiens	inactive	inactive	inactive	5
CHEMBL1900	Aldose reductase	Homo sapiens	inactive	inactive	inactive	6
CHEMBL249	Neurokinin 1 receptor	Homo sapiens	inactive	inactive	inactive	7
CHEMBL3130	PI3-kinase p110-delta subunit	Homo sapiens	inactive	inactive	inactive	6
CHEMBL275	Phosphodiesterase 4B	Homo sapiens	inactive	inactive	inactive	6
CHEMBL261	Carbonic anhydrase I	Homo sapiens	inactive	inactive	inactive	6
CHEMBL1860	Thyroid hormone receptor alpha	Homo sapiens	inactive	inactive	inactive	7
CHEMBL2414	C-C chemokine receptor type 4	Homo sapiens	inactive	inactive	inactive	7
CHEMBL315	Alpha-1b adrenergic receptor	Rattus norvegicus	inactive	inactive	inactive	7
CHEMBL5192	Botulinum neurotoxin type A	Clostridium botulinum	inactive	inactive	both	6

CHEMBL5145	Serine/threonine-protein kinase B-raf	Homo sapiens	inactive	inactive	inactive	7.5
CHEMBL2617	Tryptase beta-1	Homo sapiens	inactive	inactive	inactive	6
CHEMBL3247	HMG-CoA reductase	Rattus norvegicus	inactive	inactive	inactive	6
CHEMBL3535	Phosphodiesterase 9A	Homo sapiens	inactive	inactive	inactive	6
CHEMBL2830	Voltage-gated L-type calcium channel alpha-1C subunit	Oryctolagus cuniculus	inactive	inactive	both	5
CHEMBL2096680	Glutamate NMDA receptor; Grin1/Grin2a	Rattus norvegicus	inactive	inactive	inactive	5
CHEMBL3910	11-beta-hydroxysteroid dehydrogenase 1	Mus musculus	inactive	inactive	inactive	6
CHEMBL4662	Proteasome Macropain subunit MB1	Homo sapiens	inactive	inactive	inactive	6
CHEMBL260	MAP kinase p38 alpha	Homo sapiens	inactive	inactive	inactive	7.5
CHEMBL274	C-C chemokine receptor type 5	Homo sapiens	inactive	inactive	inactive	7
CHEMBL248	Leukocyte elastase	Homo sapiens	inactive	inactive	inactive	6
CHEMBL1901	Cholecystokinin A receptor	Homo sapiens	inactive	inactive	inactive	7
CHEMBL1929	Xanthine dehydrogenase	Homo sapiens	empty	inactive	inactive	6
CHEMBL2094108	Protein farnesyltransferase	Homo sapiens	inactive	inactive	inactive	6
CHEMBL1907603	Glutamate NMDA receptor; GRIN1/GRIN2B	Homo sapiens	inactive	inactive	inactive	5
CHEMBL2111367	PI3-kinase p110-alpha/p85-alpha	Homo sapiens	inactive	inactive	inactive	6
CHEMBL4361	Induced myeloid leukemia cell differentiation protein Mcl-1	Homo sapiens	inactive	inactive	inactive	6
CHEMBL3368	Glucocorticoid receptor	Rattus norvegicus	inactive	inactive	both	7
CHEMBL3712907	Transmembrane domain-containing protein TMIGD3	Homo sapiens	inactive	inactive	inactive	6

CHEMBL4822	Beta-secretase 1	Homo sapiens	inactive	inactive	inactive	6
CHEMBL5282	Cytochrome P450 2A6	Homo sapiens	inactive	inactive	inactive	6
CHEMBL213	Beta-1 adrenergic receptor	Homo sapiens	inactive	inactive	inactive	7
CHEMBL3142	DNA-dependent protein kinase	Homo sapiens	inactive	inactive	inactive	7.5
CHEMBL3616354	Branched-chain-amino-acid aminotransferase, mitochondrial	Homo sapiens	inactive	inactive	both	6
CHEMBL1744525	Nicotinamide phosphoribosyltransferase	Homo sapiens	inactive	inactive	inactive	6
CHEMBL5645	Cysteinyl leukotriene receptor 1	Cavia porcellus	inactive	inactive	inactive	7
CHEMBL5137	Metabotropic glutamate receptor 2	Homo sapiens	empty	inactive	both	7
CHEMBL5888	Mitogen-activated protein kinase kinase kinase 14	Homo sapiens	inactive	inactive	inactive	7.5
CHEMBL3023	Sphingosine kinase 2	Homo sapiens	inactive	inactive	inactive	6
CHEMBL2329	Dihydrofolate reductase	Candida albicans	empty	inactive	inactive	6.5
CHEMBL3108640	Bromodomain-containing protein 9	Homo sapiens	inactive	inactive	inactive	6
CHEMBL3157	Bradykinin B2 receptor	Homo sapiens	inactive	inactive	inactive	7
CHEMBL1250375	NADPH oxidase 4	Homo sapiens	inactive	inactive	inactive	6
CHEMBL206	Estrogen receptor alpha	Homo sapiens	inactive	inactive	inactive	7
CHEMBL3816	Cytosolic phospholipase A2	Homo sapiens	inactive	inactive	inactive	6
CHEMBL4296301	Reverse transcriptase protein	Human immunodeficiency virus 1	inactive	inactive	inactive	6
CHEMBL2189117	Polycomb protein EED	Homo sapiens	inactive	inactive	inactive	6
CHEMBL1781	DNA topoisomerase I	Homo sapiens	inactive	inactive	inactive	6
CHEMBL210	Beta-2 adrenergic receptor	Homo sapiens	inactive	inactive	inactive	7
CHEMBL238	Dopamine transporter	Homo sapiens	inactive	inactive	inactive	6

CHEMBL2424504	Lysine-specific demethylase 5A	Homo sapiens	inactive	inactive	inactive	6
CHEMBL5493	Free fatty acid receptor 2	Homo sapiens	inactive	inactive	inactive	6.5
CHEMBL402	HMG-CoA reductase	Homo sapiens	inactive	inactive	inactive	6
CHEMBL3753	Glutamate receptor ionotropic, AMPA 1	Rattus norvegicus	inactive	inactive	inactive	5
CHEMBL2882	Histamine H2 receptor	Cavia porcellus	inactive	inactive	inactive	7
CHEMBL1255150	G-protein coupled bile acid receptor 1	Mus musculus	inactive	inactive	inactive	7
CHEMBL1163116	Cholesteryl ester transfer protein	Oryctolagus cuniculus	inactive	inactive	inactive	5
CHEMBL4029	Interleukin-8 receptor A	Homo sapiens	inactive	inactive	inactive	6.5
CHEMBL4015	C-C chemokine receptor type 2	Homo sapiens	inactive	inactive	inactive	7
CHEMBL1804	Somatostatin receptor 2	Homo sapiens	inactive	inactive	inactive	7
CHEMBL3975	Fructose-1,6-bisphosphatase	Homo sapiens	inactive	inactive	inactive	6
CHEMBL211	Muscarinic acetylcholine receptor M2	Homo sapiens	inactive	inactive	inactive	7
CHEMBL3137262	LSD1/CoREST complex	Homo sapiens	empty	inactive	inactive	6
CHEMBL205	Carbonic anhydrase II	Homo sapiens	empty	inactive	inactive	6
CHEMBL3815	Squalene synthetase	Rattus norvegicus	empty	inactive	inactive	6
CHEMBL4439	TGF-beta receptor type I	Homo sapiens	inactive	inactive	inactive	7.5
CHEMBL3356	Cytochrome P450 1A2	Homo sapiens	inactive	inactive	inactive	6
CHEMBL1907601	Cyclin-dependent kinase 4/cyclin D1	Homo sapiens	inactive	inactive	inactive	7.5
CHEMBL3385	MAP kinase ERK1	Homo sapiens	inactive	inactive	inactive	6.5
CHEMBL1907605	Cyclin-dependent kinase 2/cyclin E1	Homo sapiens	inactive	inactive	inactive	7.5
CHEMBL3959	Quinone reductase 2	Homo sapiens	inactive	inactive	inactive	6

CHEMBL3024	Serine/threonine-protein kinase PLK1	Homo sapiens	inactive	inactive	inactive	7.5
CHEMBL5441	Dihydrofolate reductase	Escherichia coli	inactive	inactive	inactive	6
CHEMBL4777	Neuropeptide Y receptor type 1	Homo sapiens	inactive	inactive	inactive	7
CHEMBL1829	Histone deacetylase 3	Homo sapiens	inactive	inactive	inactive	6
CHEMBL3623	Quinone reductase 1)	Homo sapiens	inactive	inactive	inactive	6
CHEMBL228	Serotonin transporter	Homo sapiens	inactive	inactive	inactive	6
CHEMBL1949	Cyclophilin A	Homo sapiens	empty	inactive	both	6
CHEMBL4414	Plasmepsin 2	Plasmodium falciparum	inactive	inactive	inactive	6
CHEMBL4372	Anthrax lethal factor	Bacillus anthracis	inactive	inactive	inactive	6
CHEMBL1907604	Glutamate NMDA receptor; GRIN1/GRIN2A	Homo sapiens	inactive	inactive	inactive	6.5
CHEMBL3437	Amine oxidase, copper containing	Homo sapiens	inactive	inactive	inactive	6
CHEMBL4358	Arachidonate 15-lipoxygenase	Oryctolagus cuniculus	inactive	inactive	inactive	6
CHEMBL2111389	CDK9/cyclin T1	Homo sapiens	inactive	inactive	inactive	7.5
CHEMBL1977	Vitamin D receptor	Homo sapiens	empty	inactive	inactive	7
CHEMBL3594	Carbonic anhydrase IX	Homo sapiens	inactive	inactive	inactive	6
CHEMBL5443	Cell division cycle 7-related protein kinase	Homo sapiens	inactive	inactive	both	7.5
CHEMBL2489	Prostanoid EP4 receptor	Mus musculus	empty	inactive	inactive	7
CHEMBL3797	Serine-protein kinase ATM	Homo sapiens	inactive	inactive	inactive	6.5
CHEMBL203	Epidermal growth factor receptor erbB1	Homo sapiens	inactive	inactive	inactive	7.5
CHEMBL217	Dopamine D2 receptor	Homo sapiens	inactive	inactive	inactive	7
CHEMBL3807	T-cell protein-tyrosine phosphatase	Homo sapiens	empty	inactive	both	6

CHEMBL4198	Inhibitor of apoptosis protein 3	Homo sapiens	inactive	inactive	inactive	6
CHEMBL1792	Somatostatin receptor 5	Homo sapiens	inactive	inactive	inactive	7

The entry corresponding to Mycobacterium tuberculosis is highlighted in bold

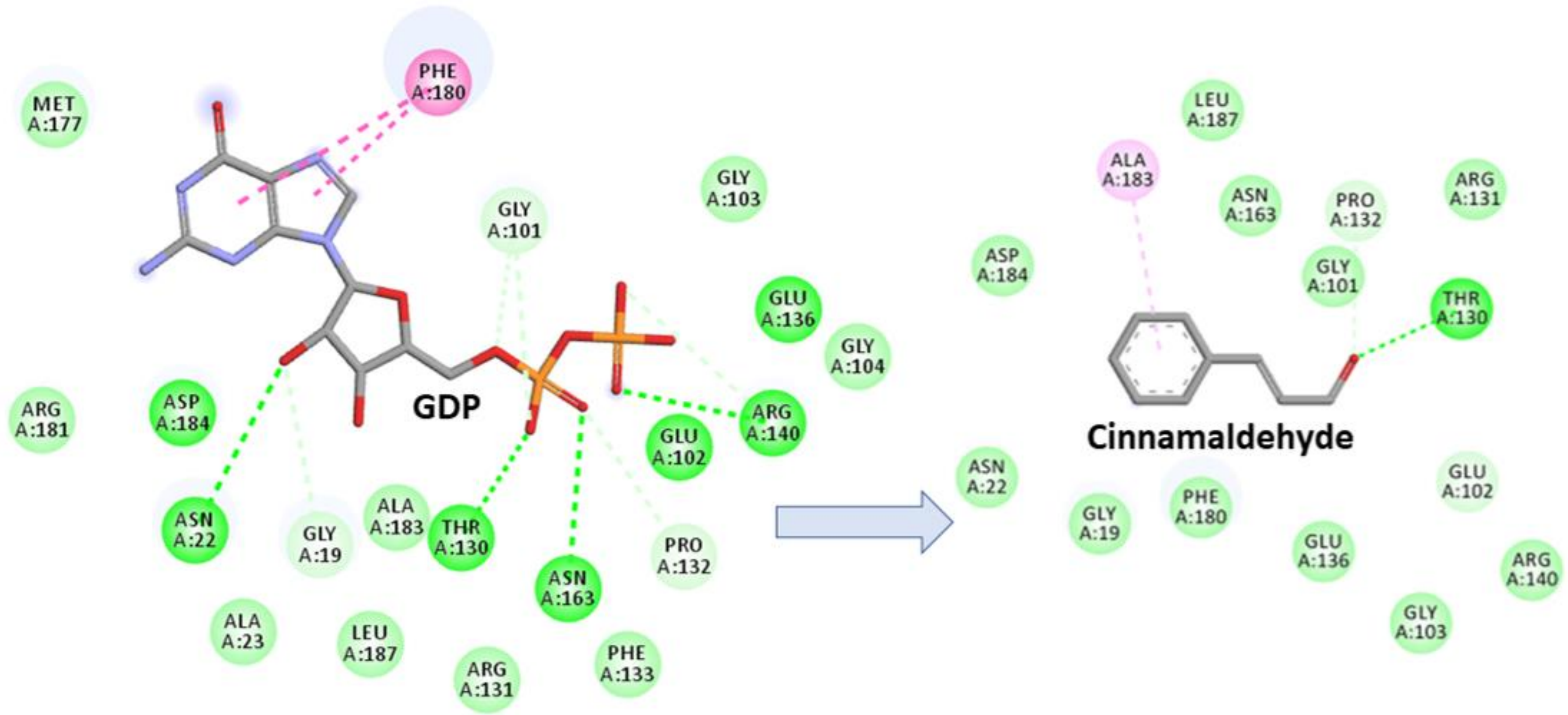

Figure S1. This is a figure of the binding modes for the best poses of the docked FtsZ complexes with GDP nucleotide moiety and cinnamaldehyde in the active site.

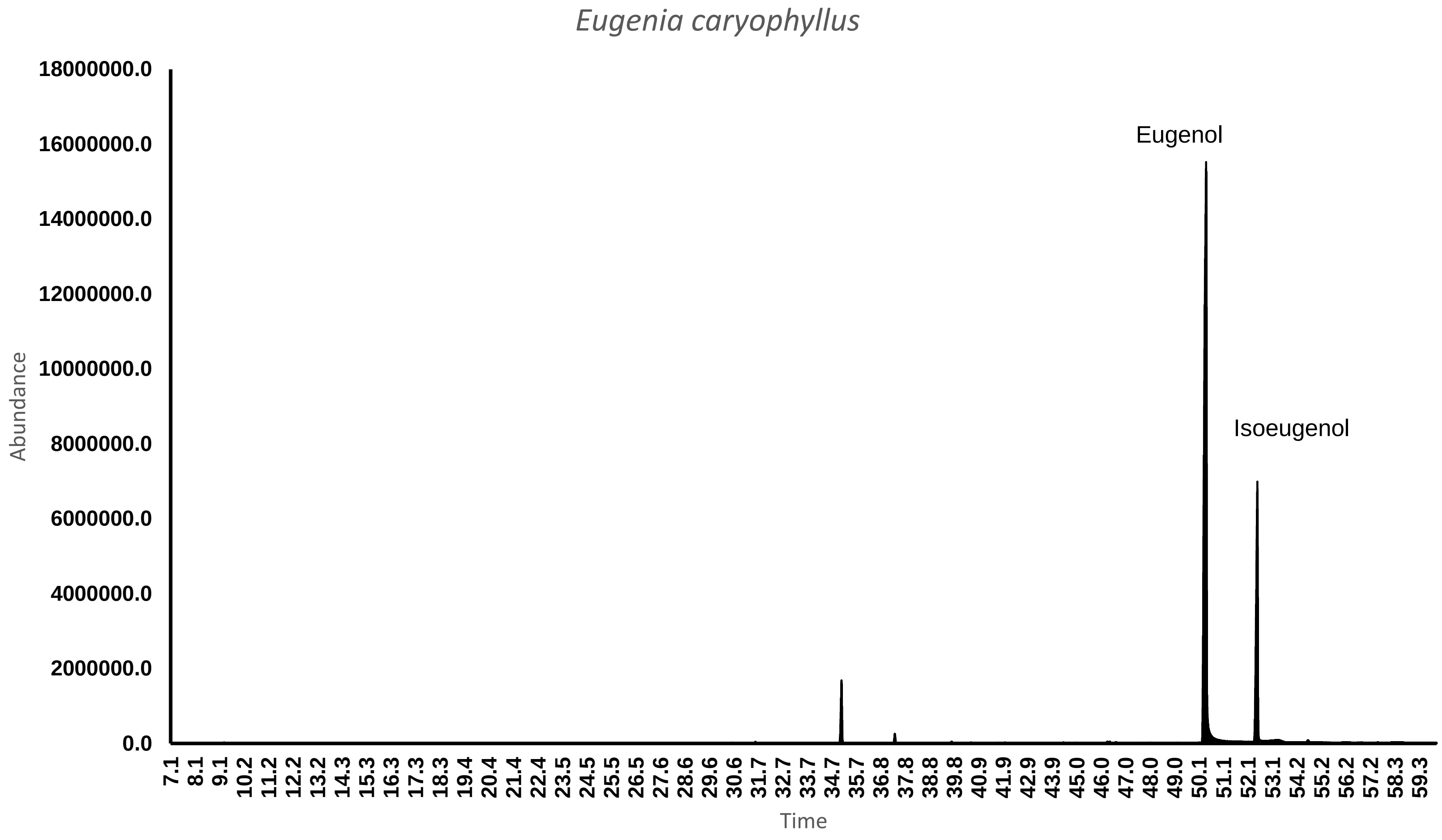

Figure S2. This is the gas chromatogram of *Eugenia caryophyllus* essential oil showing eugenol as the dominant compound.

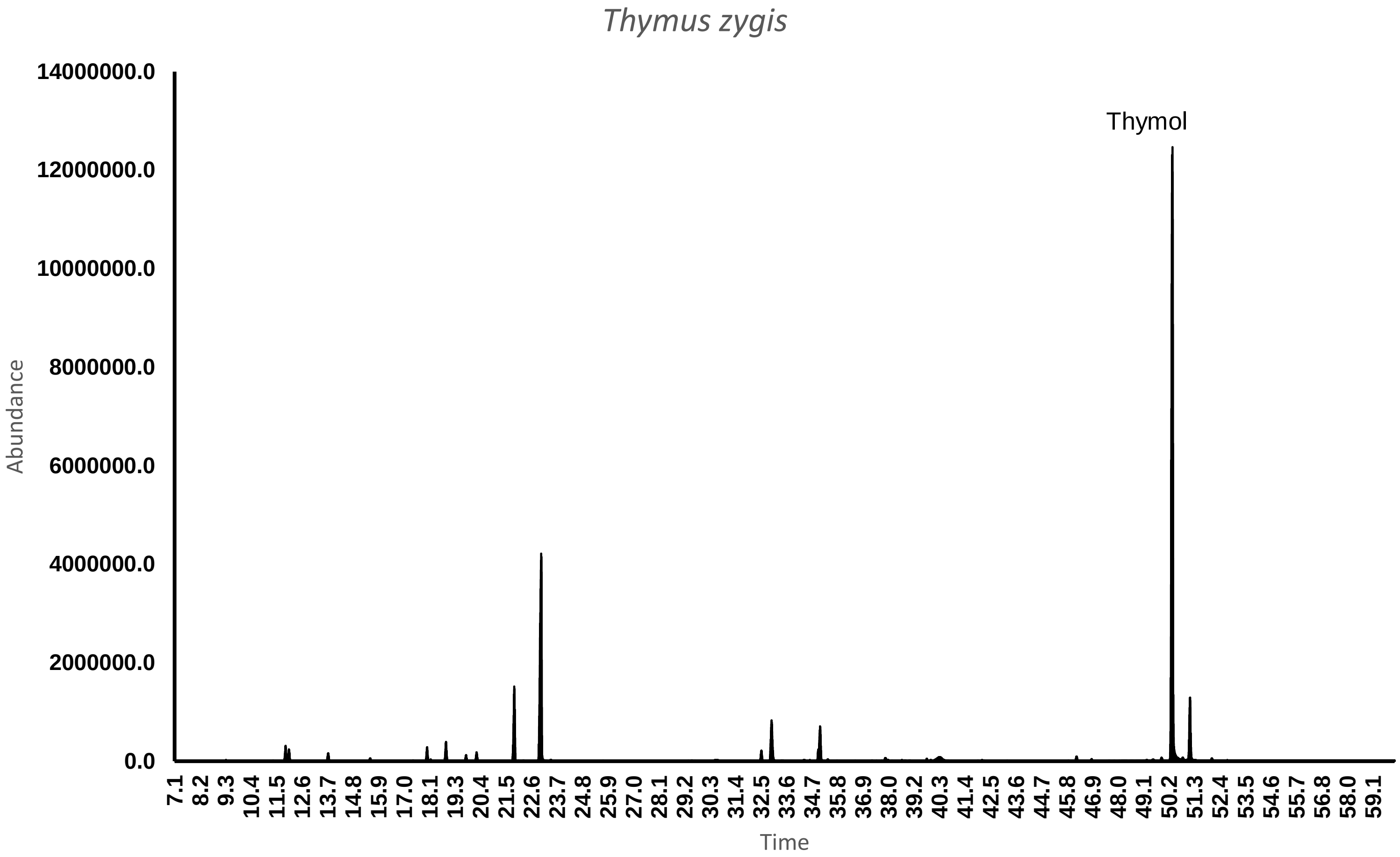

Figure S3. This is the gas chromatogram of *Thymus zygis* showing thymol as a major compound

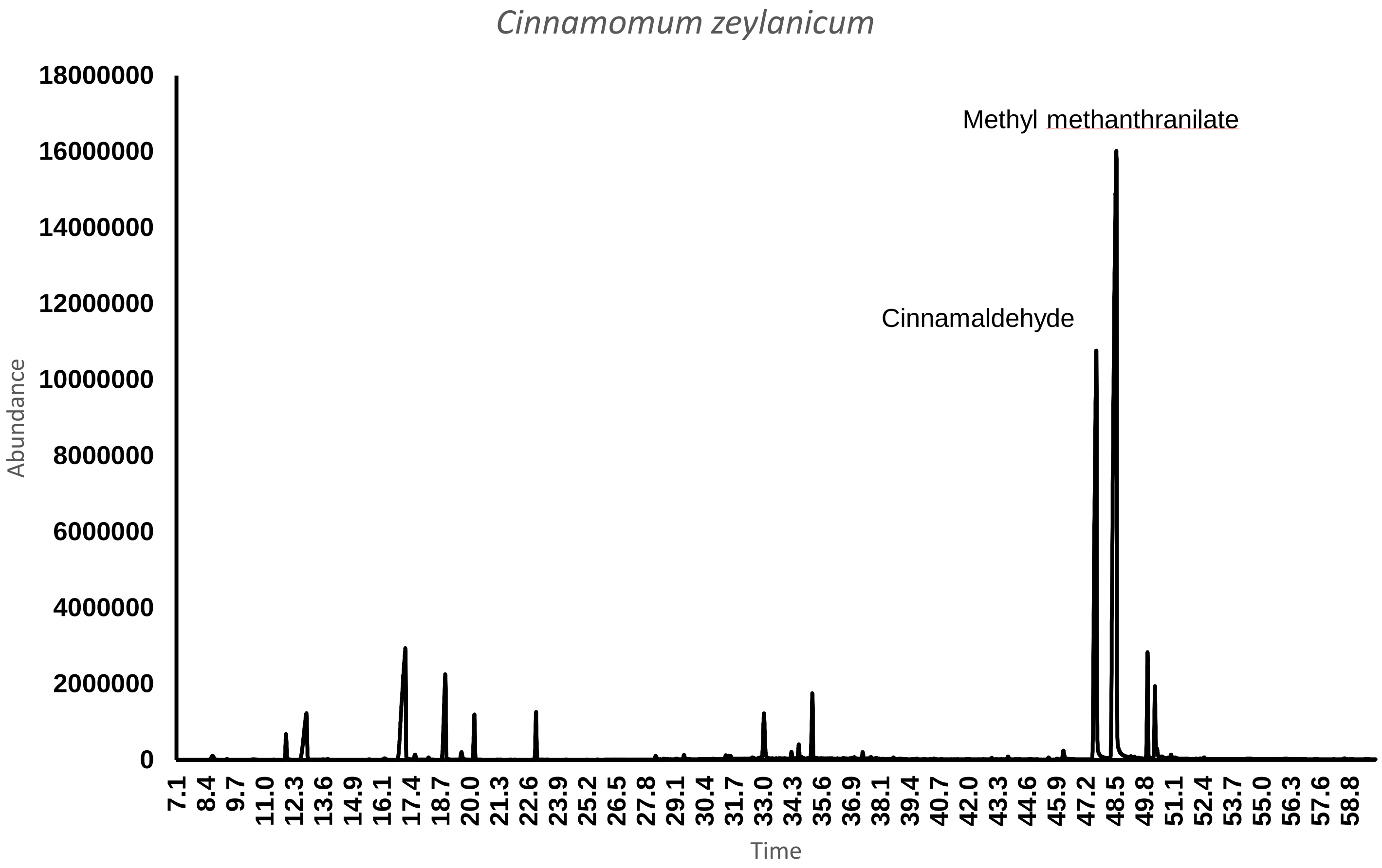

Figure S4. This is the gas chromatograms of essential oils of *Cinnamomum zeylanicum* (bark) showing cinnamaldehyde as the major compounds
